# How exercise adherence affects emotion regulation in Chinese college students: The chain mediating effect of sleep quality and self-efficacy

**DOI:** 10.1371/journal.pone.0338572

**Published:** 2026-01-08

**Authors:** Guowei Wang, Zeyuan Wang, Fanzheng Mu, Guolin Li, Bo Li

**Affiliations:** 1 Henan Sport University, Zhengzhou, China; 2 School of Physical Education, Zhengzhou University (Main Campus), Zhengzhou, China; 3 School of Physical Education Science, Fujian Normal University, Fuzhou, China; 4 Institute of Sports Science, Nantong University, Nantong, China; University of Rijeka Faculty of Health Studies: Sveuciliste u Rijeci Fakultet zdravstvenih studija, CROATIA

## Abstract

**Objective:**

This study investigated the mechanism through which exercise adherence enhances emotional regulation ability in college students, focusing on the chain mediating pathway involving sleep quality and self-efficacy. It provides a theoretical foundation for exercise-based interventions to promote psychological health in this population.

**Methods:**

A cross-sectional design was employed, with 8,899 chinese college students from Henan Province selected via stratified cluster sampling. Data were collected using the Exercise Adherence Scale (EAS), Emotional Intelligence Scale (EIS), Pittsburgh Sleep Quality Index (PSQI), and General Self-Efficacy Scale (GSES). Statistical analyses were conducted using EXCEL, Mplus 8.3, and SPSS 27.0.1.

**Results:**

Exercise adherence exerted a significant direct positive effect on emotional regulation ***(β = 0.289,56.67% of the total effect)***. Significant independent mediating effects were observed for sleep quality ***(β = 0.010,6.33%)*** and self-efficacy***(β = 0.200,89.59%)***. Crucially, a chain mediation pathway was identified: exercise adherence improved sleep quality, which subsequently enhanced self-efficacy, ultimately strengthening emotional regulation ability***(β = 0.010,4.52%)***.

**Conclusion:**

Exercise adherence directly enhances emotional regulation and indirectly influences it through the “sleep quality → self-efficacy” chain pathway. These findings reveal a synergistic multi-path mechanism and provide empirical support for designing targeted exercise-based mental health interventions.

## Introduction

The 2024 National Survey of College Student Mental Health indicates a dose-dependent reduction in depression and anxiety risks among Chinese college students with increased physical activity frequency [1]. Emotional Regulation (ER) refers to the process by which individuals influence and modulate their emotional responses. It operates by regulating the underlying systems that generate emotions, such as attentional mechanisms, cognitive appraisal, and physiological activation. The primary functions of emotional regulation include facilitating the pursuit of goals, enhancing subjective well-being, and supporting the healthy development of personality [2].

In recent years, mental health issues among chinese college students have garnered increasing societal attention, and emotional regulation has emerged as a critical indicator of psychological adaptation [3], Emotion regulation plays a crucial role in mental health, as it significantly influences an individual’s academic performance, interpersonal relationships, and overall quality of life. Numerous studies have indicated that depression and anxiety are prevalent among college students [4], Emotional regulation is thought to evolve progressively from late adolescence into early adulthood [5], The statement aligns with the developmental timeline of both the physical and mental growth of college students [6,7], At the same time, this process also involves an individual’s regulation of their own emotions, experiences, and expressions [8,9]. Regular physical exercise is widely recognized for its multifaceted benefits on physical and psychological well-being. However, despite these established advantages, a significant proportion of college students fail to maintain adequate exercise adherence (EA), often characterized by low participation rates, irregular engagement [10], A robust association has been established between exercise adherence and emotional regulation capacity in college students, with bidirectional interactions observed between these variables [11,12].

Sleep Quality (SQ) is a critical determinant of an individual’s overall health and emotional regulation. Chinese college students often encounter the challenge of deteriorating sleep quality, which may be attributed to various behavioral and psychological factors, including irregular physical exercise and low self-efficacy [13]. Research indicates that the relationship between sleep disorders and both depression and anxiety is bidirectional [14], for instance, insomnia can exacerbate emotional dysregulation and negative affective biases, which are core features of depression and anxiety. Conversely, the hyperarousal and ruminative processes characteristic of these mood disorders can intrude upon sleep, disrupting sleep initiation and maintenance. Furthermore, physical activity has been shown to have effects comparable to those of antidepressant treatments, proving beneficial in the management of depression [15], Additionally, high-quality sleep can significantly enhance emotional regulation capabilities [16]. This is posited to occur through several key mechanisms, including the restoration of prefrontal cortex function for top-down emotional control, the reduction of amygdala reactivity to negative stimuli, and the overnight consolidation of emotional memories, which together facilitate more adaptive responses to next-day emotional challenges. Empirical evidence confirms a bidirectional depression-sleep disorder comorbidity [17]. This reciprocal relationship can be explained by shared neurobiological pathways: sleep disruptions dysregulate the hypothalamic-pituitary-adrenal (HPA) axis and monoamine systems, thereby fostering depressive symptoms; conversely, the rumination and hyperarousal inherent to depression disrupt sleep architecture and circadian rhythms, perpetuating a vicious cycle.

Among college populations, sleep quality is frequently compromised, with sociodemographic factors, sleep hygiene practices, residential environment, and substance use identified as key determinants [18]. Low-intensity physical activity predominates in this demographic, while sleep deficiency remains prevalent. Research suggests that for optimal mental health, a combination of moderate-to-vigorous physical activity and adequate sleep is particularly effective [19], However, when the specific goal is to improve sleep quality, evidence indicates that moderate-intensity exercise may be more beneficial than vigorous activity [20]. Theoretically, sleep quality modulates emotional regulation capacity through neurocognitive pathways [21], and MVPA-sleep interactions correlate significantly with psychopathology incidence in collegiate cohorts [22].

Self-efficacy, conceptualized within Bandura’s social cognitive theory [23], denotes individuals’ confidence in mobilizing competencies to attain goals [24]. Empirical studies demonstrate that moderate-intensity exercise enhances college students’ physical health through SE elevation [25]. Crucially, SE mediates the physical activity-quality of life association, particularly in clinical populations. This mediating pathway operates via improved treatment adherence, including overcoming disease-specific barriers to exercise participation [26]. Collegiate populations — undergoing critical socialization transitions — demonstrate elevated prevalence of anxiety and depression when navigating academic demands and interpersonal challenges [27], with consequent quality of life reductions. Crucially, physical exercise behavior significantly predicts both negative affect mitigation and self-efficacy enhancement [28], Increased exercise participation elevates SE levels, reinforcing perceived competence and augmenting emotion regulatory capacity during distress exposure. This sequential pathway Exercise adherence → SE augmentation → Negative affect reduction empirically establishes SE’ s mediating role in psychological adjustment mechanisms while providing evidence-based support for exercise interventions in campus mental health programming [29].

This study operationalizes sleep quality and self-efficacy as mediators based on three theoretically grounded considerations: (1) Empirically established linkages: Significant correlations previous among EA, ER, SQ, and SE. (2) Limitations of single-mediator approaches: Previous research predominantly examines isolated mediating pathways, overlooking potential mediator synergies. (3) Mechanistic complexity requirement: A chain mediation model: EA → SQ → SE → ER is proposed to elucidate the sequential psychophysiological pathway through which physical activity influences ER. Therefore, the following hypotheses are put forward:

H1: EA, SQ, SE, and ER exhibit significant intercorrelations.
H2: SQ and SE independently mediate the EA-ER relationship.
H3: SQ and SE have a chain mediating effect in EA and ER.

**Fig 1:**
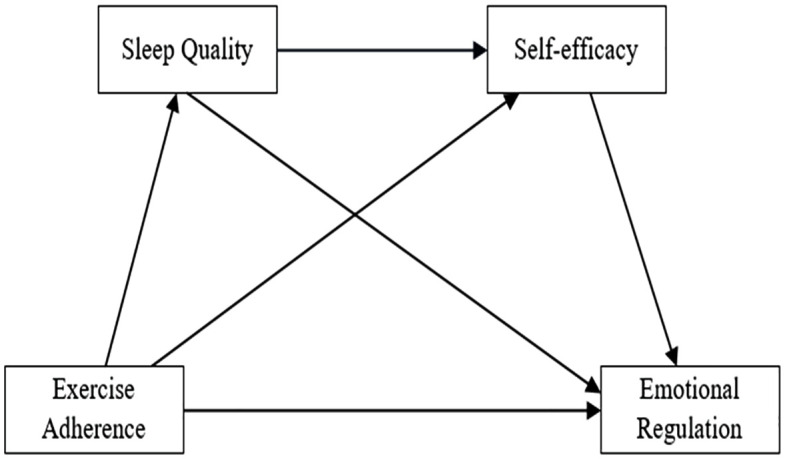
Hypothetical model diagram.

The proposed conceptual model is presented in Fig 1.

## Materials and methods

### Subjects

#### Ethics statement.

The study protocol was approved by the ethics committee of Nantong University 2022(70). Before formal investigation and testing, the researchers received the informed consent of the subjects involved in this study.

#### Informed consent.

We are honored to deliver this questionnaire to you. As the youth of contemporary China, you are the future and hope of our country. We want to take this opportunity to understand your physical fitness and exercise habits. By gathering feedback on your current status, we will develop targeted strategies for promoting the health of college students. The above content serves as the introductory part of the survey. We have obtained informed consent from all participants. Informed consent was obtained prior to participation. Participants were instructed that by proceeding with the questionnaire after reading the introductory statement, they were providing their consent. They were also informed that they were free to decline participation by simply closing the questionnaire.

Henan Province was selected as a representative Central China region with substantial higher education resources to control for geographical confounders. A cross-sectional design was employed, with 8,899 undergraduates recruited from higher education institutions in Henan Province through stratified cluster sampling. Participants comprised full-time undergraduates excluding postgraduate students from Ministry of Education-accredited higher education institutions in Henan Province, China. A stratified cluster sampling approach was implemented across three socioeconomic tiers: High-development tier: Zhengzhou, Medium-development tier: Xinyang, Luoyang, and Xinxiang, Lower-development tier: Shangqiu, Kaifeng, and Jiaozuo. The recruitment period for the study spanned from 08/10/2024–09/11/2024, with the subsequent questionnaire survey being conducted from 11/11/2024–24/11/2024.

Institutional selection prioritized: (1) nationally accredited higher education institutions (including vocational colleges); (2) demographic representativity across age, enrollment size, and academic standing; and (3) administrative capacity for longitudinal monitoring. Participants were stratified by gender (male/female) and academic year (Years 1–4), including 5th year students majoring in medicine. with final sample distributions detailed in Table 1.

**Table 1:** List of the distribution of research subjects.

Variable		n	Percentage
Gender			
	Male	3172	35.6
	Female	5752	64.4
Ethnicity			
	Han ethnicity	8736	98.2
	Ethnic minorities	163	1.8
Grade			
	Freshman	6464	72.6
	Sophomore	2107	23.7
	Junior	282	3.2
	Senior	46	0.5
	Total	8899	100

The minimum required sample size was calculated using Formula. As shown in Formula 1, with α (Type I error rate) = 0.05, δ (allowable error) = 0.01, and ρ (sample proportion) = 0.05. Given a finite population size (N) of 195,949 (official demographic registry data), the calculation yielded a minimum sample of 1,808 participants [30].


$$n=\frac{\frac{Z_{\alpha }^{\text{2}}*\sigma^{\text{2}}}{\delta^{\text{2}}}}{\text{1}+\frac{Z_{\alpha }^{\text{2}}*\sigma^{\text{2}}}{\delta^{\text{2}}}/N}$$ (1)

Quality Control Procedures: Standardized protocols were implemented across all phases. Pre-survey training: Investigators received centralized training on research protocols and standardized administration scripts. Response validation: Questionnaires were excluded if they met any of the following criteria: Unidentifiable institution affiliation, Uniform response patterns (*e.g., identical selections throughout*); Systematic responding (*e.g., straight-line sequences: 1-2-3-4-5*); Analytical rigor: Statistical methods were selected based on variable properties and distributional assumptions per contemporary reporting standards. Ultimately, 8,889 validated responses were retained for analysis.

### Scales

(1) Exercise Adherence Scale (EAS)

The study utilized the EAS developed by Gu [31]. The EAS comprises three dimensions: exercise behavior, effort commitment, and emotional experience, encompassing a total of 14 items. It employs a five-point scoring system, with higher scores indicating greater exercise persistence. The scale demonstrates strong reliability (0.947) and favorable fit indices (χ²/df = 2.896, CFI = 0.945, GFI = 0.901, RMSEA = 0.069), suggesting its good reliability and validity, and making it suitable as a general research instrument. In this study, the test-retest reliability was 0.867, and its applicability among Chinese college students has been confirmed in multiple studies.

(2) Emotional Intelligence Scale (EIS)

Emotional regulation (ER) capacity was assessed using the 8-item Emotion Regulation subscale of the EIS translated and validated for Chinese populations by Wang [32]. Originally developed by Scott et al. based on Mayer and Salovey’s (1990) theoretical framework, the full EIS comprises 33 items across four domains. The selected subscale (Items 2,6,7,10,12,14,21,28) employs a 5-point Likert scale (1 = Very inconsistent; 5 = Very consistent), with higher scores indicating superior ER capacity. In the current sample, internal consistency was acceptable (Cronbach’s α = 0.809), consistent with previous validation studies in Chinese collegiate populations [33–36].

(3) Pittsburgh Sleep Quality Index (PSQI)

This study assessed the sleep quality of chinese college students using the Pittsburgh Sleep Quality Index (PSQI), revised and adapted by Liu [37].The PSQI comprises 19 items across 7 dimensions, each scored from 0 to 3. The total score ranges from 0 to 21,with higher scores indicating poorer sleep quality [38], The scale uses reverse scoring, consistent with this interpretation. Its applicability for Chinese college students is well-established [39,40], The Cronbach’s alpha coefficient for internal consistency reliability in this study was 0.845 [41–43].

(4) General Self-Efficacy Scale(GSES)

In this study, the General Self-Efficacy Scale (GSES) translated and revised by Wang was used to measure general self-efficacy in college students [44]. Originally developed by Schwarzer, the GSES has demonstrated good reliability through analyses by Chinese scholars, with a Cronbach’s α coefficient of 0.730. The scale comprises 10 items rated on a 4-point Likert scale (1–4). Total scores represent overall self-efficacy levels, with higher scores indicating greater self-efficacy. Its applicability among Chinese college students is well-established [45–50].

### Statistical methods

Data processing and analysis were conducted using Excel for data entry and cleaning, Origin 2021, and Mplus 8.3. The analytical procedure consisted of the following steps: First, data preprocessing was performed. Invalid questionnaires were identified and excluded based on predetermined screening criteria unidentifiable institution affiliation, uniform response patterns, or systematic straight-line responding. Second, to assess the potential threat of common method bias, Harman’s single-factor test was conducted on all scale items in SPSS. The unrotated exploratory factor analysis revealed thirteen factors with eigenvalues greater than 1. The first factor accounted for 33.4% of the total variance, which is below the critical threshold of 40%, indicating that common method bias was not a serious concern in this study [51], Third, descriptive statistics (means, standard deviations) and one-way analyses of variance were computed for the main study variables EA, ER, SQ, SE across demographic groups gender, ethnicity, grade using SPSS. Fourth, zero-order Pearson correlation analyses were performed in Origin 2021 to examine the bivariate relationships among the core variables: exercise adherence, emotional regulation, sleep quality, and self-efficacy, as well as key demographic covariates (gender, age, grade). Finally, to test our primary hypotheses regarding mediation, a chain mediation model was specified and analyzed using Mplus 8.3. The model posited that exercise adherence (EA) influences emotional regulation (ER) through three pathways: the direct path (EA → ER), and the indirect paths via sleep quality (SQ) and self-efficacy (SE), including the chain pathway (EA → SQ → SE → ER). All mediator and outcome variables were regressed onto the covariates of gender, age, and grade to control for their potential confounding effects.

The model parameters were estimated using the maximum likelihood estimator. To robustly test the significance of the specific indirect effects, we employed the bias-corrected nonparametric bootstrap method with 5,000 resamples. This procedure generates an empirical approximation of the sampling distribution for each indirect effect, from which 95% bias-corrected confidence intervals are derived. An indirect effect is considered statistically significant at the α = 0.05 level if its 95% bootstrap confidence interval does not contain zero.

## Results

### Analysis of descriptive results

Table 2 shows descriptive analysis revealed that college students’ EA scores were 53.050 ± 9.437. Significant differences were observed based on gender ***(P < 0.001, η² = 0.045)*** and grade ***(P < 0.001)***. Gender accounted for 4.5% of the variance in EA scores. No significant ethnic differences were found. ER scores were 29.660 ± 4.874, with significant differences by gender ***(P < 0.001)*** and grade ***(P < 0.001, η² = 0.002)***. Grade explained only 0.2% of the variance in ER. No significant ethnic differences were observed. SQ scores were 2.080 ± 0.814, differing significantly by gender ***(P < 0.001)*** and grade ***(P < 0.001, η² = 0.007)***. The variance explained by grade for SQ was minimal 0.7%. Ethnic differences were non-significant. SE scores were 18.370 ± 5.662, showing a significant gender difference ***(P < 0.001, η² = 0.008)***, which accounted for 0.8% of the variance. SE was the only variable unaffected by grade ***(P > 0.05)***, and no significant ethnic differences were found. These findings suggest the presence of gender-specific mechanisms influencing these variables.

**Table 2:** Descriptive analysis of the main research variables.

			EA	ER	SQ	SE
Gender						
	Male					
		M	53.050	29.660	2.080	18.370
		sd	9.437	4.874	0.814	5.662
	Female					
		M	49.180	29.530	1.990	17.410
		sd	7.950	4.071	0.781	4.954
	***F***		96.581	82.380	21.229	33.233
	***P***		<0.001	<0.001	<0.001	<0.001
	***η*** ^***2***^		0.045	<0.010	0.002	0.008
Ethnic						
	Han ethnicity					
		M	50.560	29.570	2.020	17.760
		sd	8.710	4.378	0.795	5.242
	Ethnic minorities					
		M	50.580	29.960	2.020	17.560
		sd	8.673	4.130	0.728	4.986
	***F***		0.120	2.106	2.693	0.632
	***P***		0.729	0.147	0.101	0.427
	***η*** ^***2***^		<0.001	<0.001	<0.001	<0.001
Grade						
	Freshman					
		M	50.650	29.690	1.990	17.650
		sd	8.588	4.283	0.790	5.206
	Sophomore					
		M	49.880	29.210	2.110	17.940
		sd	8.858	4.566	0.799	5.299
	Junior					
		M	53.050	29.641	2.180	18.590
		sd	9.868	4.721	0.798	5.338
	Senior					
		M	54.830	30.430	2.390	18.780
		sd	6.691	4.987	0.770	5.609
	***F***		15.921	6.935	19.887	4.748
	***P***		<0.001	<0.001	<0.001	0.003
	***η*** ^***2***^		0.005	0.002	0.007	0.002

Note: EA = Exercise adherence

ER = Emotional Regulation

SQ = Sleep Quality

SE = Self-efficacy

### Correlation analysis

**Fig 2:**
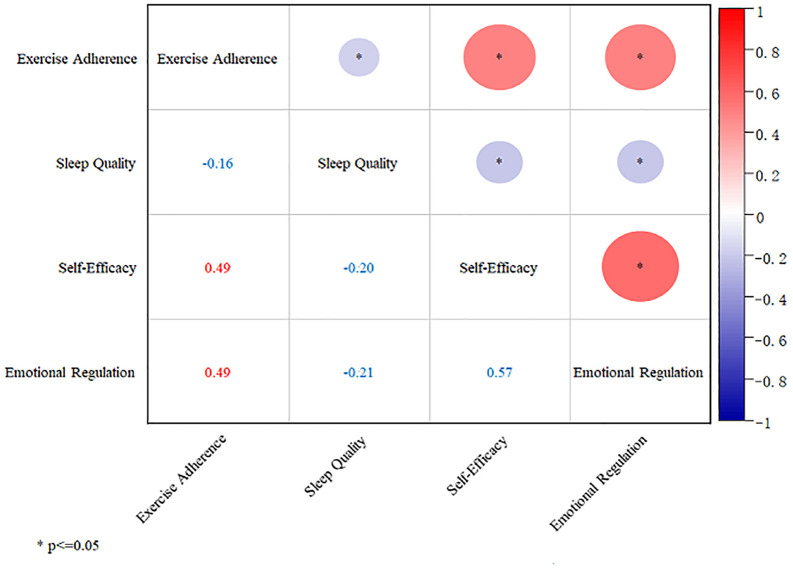
Correlation analysis chart.

Fig 2 presents variable correlation results. Due to reversed SQ scoring: EA negatively correlated with SQ ***(r = −0.160, P < 0.05)***, indicating higher exercise consistency associates with better sleep quality. EA positively correlated with ER ***(r = 0.490, P < 0.05)*** and SE ***(r = 0.490, P < 0.05)***. ER negatively correlated with SQ ***(r = −0.210, P < 0.05)***, reflecting better sleep quality relates to higher emotion regulation. ER positively correlated with SE ***(r = 0.570, P < 0.05)***. SQ negatively correlated with SE ***(r = −0.200, P < 0.05)***, showing better sleep quality associates with higher self-efficacy.

### Regression analysis

Table 3 displays regression results: EA significantly predicted ER ***(β = 0.145, P < 0.001; R² = 0.394)***. With reversed SQ scoring, EA negatively predicted SQ ***(β = −0.017, P < 0.001; R² = 0.042)***. EA positively predicted SE ***(β = 0.284, P < 0.001; R² = 0.261)***

**Table 3:** Regression analysis of each variable in the model.

	Regression equation		Overall fitting index			Significance of the regression coefficient		
Result variable			*R*	*R* ^*2*^	*F*	*β*	*SE*	*t*
ER			0.628	0.394	964.818^***^			
	EA					0.145	0.005	29.705^***^
		Gender				0.759	0.078	9.788^***^
		Age				−0.361	0.065	−5.534^***^
		Ethnic				−0.222	0.269	−0.823
SQ			0.204	0.042	96.559^***^			
	EA					−0.017	0.001	−17.296^***^
		Gender				−0.154	0.018	−8.716^***^
		Age				0.124	0.015	8.372^***^
		Ethnic				0.008	0.061	0.134
								
SE			0.511	0.261	628.330^***^			
	EA					0.284	0.006	49.848^***^
		Gender				0.048	0.103	0.467
		Age				0.405	0.086	4.701^***^
		Ethnic				0.305	0.356	0.856

Note: ^***^ in the text represents ***P < 0.001***.

### Sleep Quality and self-efficacy: A chain mediating effect test

**Fig 3:**
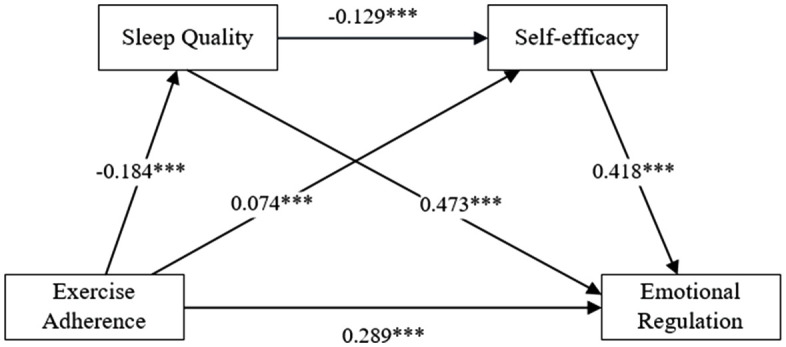
Path coefficient graph.

As shown in Fig 3, path analysis revealed EA significantly predicted ER ***(β = 0.289, P < 0.001)***; ***SQ (β = −0.184, P < 0.001)***; SE ***(β = 0.473, P < 0.001)***; SQ significantly predicted; SE ***(β = −0.129, P < 0.001)***; ER ***(β = 0.074, P < 0.001)***; SE significantly predicted ER ***(β = 0.418, P < 0.001)***

Table 4 shows the direct effect of EA on ER was ***β = 0.289 (95% CI [0.266, 0.312])***, accounting for 56.67% of the total effect. The mediating effect through SQ was ***β = 0.010 (95% CI [0.010, 0.017])***, contributing 6.33% to the total effect. Mediation via SE yielded ***β = 0.200 (95% CI [0.183, 0.213])***, representing 89.59% of the total effect. Finally, the chain mediating effect through both SQ and SE was ***β = 0.010 (95% CI [0.008, 0.012])***, explaining 4.52% of the total effect. All confidence intervals excluded zero, confirming statistical significance.

**Table 4:** Mediating effects analysis using bootstrap method.

Path	Standardized effect value	Boot SE	95% CI Lower	95% CI Upper	Effect proportion
Total effect	0.510	0.011	0.489	0.531	
Direct effect	0.289	0.012	0.266	0.312	56.67%
Total indirect effect	0.221	0.008	0.206	0.237	43.44%
EA → SQ → ER	0.010	0.002	0.010	0.017	6.33%
EA → SE → ER	0.200	0.008	0.183	0.213	89.59%
EA → SQ → SE → ER	0.010	0.001	0.008	0.012	4.52%

Note: N = 8,899. Bootstrap sample size = 5000. CI = Confidence Interval. Indirect effects are significant if the 95% bias-corrected confidence interval does not contain zero.

## Discussion

This study investigates the chain mediation of sleep quality and self-efficacy between regular exercise and emotion regulation ability in college students. Using standardized scales, we demonstrate that exercise adherence enhances emotion regulation through the sequential pathways of improved sleep quality and self-efficacy. These findings indicate that sustained exercise participation yields significant improvements in students’ emotion regulation capacity, with sleep quality and self-efficacy serving as critical mediating factors. By elucidating the integrated relationships among exercise adherence, sleep quality, self-efficacy, and emotion regulation, our research establishes a scientific foundation for designing comprehensive interventions to enhance college students’ emotional well-being.

### The direct effect of regular exercise on college students’ emotional regulation ability

Consistent exercise significantly enhances emotion regulation ability in college students, aligning with prior research [52,53], Our findings, which focus on exercise adherence, demonstrate that maintaining regular physical activity is crucial for emotional adaptability and management capacity. To further contextualize our results, previous literature suggests that the benefits of adherence may be partly explained by the effects of specific exercise types and intensities. For instance, studies have indicated that aerobic exercise can bolster all three emotion regulation stages—perception, assessment, and action—effectively diminishing negative emotional responses. Furthermore, vigorous aerobic exercise has demonstrated particular efficacy in alleviating anxiety and elevating overall emotion regulation [54,55], potentially through enhanced cognitive reappraisal of emotional contexts and strengthened control over expressive behaviors.

Despite known gender differences in emotion regulation dimensions [56], this study demonstrates that regular exercise significantly enhances emotion regulation capacity in both sexes. While prior research reported no significant correlation between physical activity levels and emotion regulation [57], potentially due to divergent exercise assessment protocols—our findings reveal robust associations. Notably, high-intensity exercise participants exhibit substantially lower depressive and anxiety symptoms than low/moderate-intensity groups, underscoring the importance of exercise persistence. Furthermore, although resistance and flexibility training benefit physical health and athletic performance, they show limited effects on emotion regulation or stress physiology. In contrast, aerobic exercise produces significant improvements in emotion regulation [58]. aligning with our results. Critically, sustained exercise buffers stress-induced physiological responses, enhancing emotional recovery [59], and effectively mitigating post-stress anger and anxiety [48].

Self-determination theory posits that intrinsic motivation and need satisfaction positively influence emotional states by fostering adaptive cognition and behavior [60]. Physical exercise driven by autonomous motivation elicits these beneficial effects. However, the benefits of exercise are contingent upon its motivational context. Research demonstrates that extrinsic or controlled motivation can lead to negative outcomes, termed sports anomie behavior [61]. Extending this logic, it follows that when physical exercise transforms into an obligatory or compulsive behavior—a manifestation of controlled motivation—the potential for negative psychological consequences arises. In such cases, the activity may no longer yield pleasure but instead foster anxiety, guilt, or other forms of psychological distress, thereby undermining its role in emotion regulation. This confirms that the emotional regulation benefits of exercise critically depend on autonomous motivation. Crucially, when physical exercise becomes a psychological burden, its physiological advantages may be negated by accompanying stress or even produce detrimental outcomes [61]. These findings demonstrate that physical exercise cannot be universally presumed to enhance emotion regulation. Critical distinctions must be made regarding exercise modality, intensity, underlying motivation, and individual differences. Research consistently reveals that the exercise-emotion regulation relationship transcends simplistic formulations. Notably, studies highlight aerobic exercise’s distinct advantages within emotion regulation mechanisms. Consequently, physical education curricula should strategically incorporate diverse aerobic activities. Future investigations should elucidate how specific movement patterns modulate core emotion regulation components—particularly attentional deployment, cognitive modification, and response adjustment.

### The mediating role of sleep quality in the ability to persist in exercise and emotional regulation ability

This study demonstrates that consistent exercise improves college students’ emotion regulation capacity through enhanced sleep quality, aligning with prior research [52]. Academic demands, interpersonal pressures, and performance expectations commonly compromise sleep quality and emotional stability in this population. Substantial evidence confirms significant associations between physical exercise and sleep quality [62,63], with most students exhibiting both insufficient exercise and poor sleep [64]. Crucially, regular exercise maintains social rhythm stability—a key determinant of sleep health. Individuals with poor sleep quality demonstrate lower social rhythm regularity than those with healthy sleep patterns, while active participants in scheduled activities including but not limited to exercise consistently report better sleep outcomes [65].

Sleep quality decline impairs cognitive control and emotion regulation capacity [66], Exercise-induced sleep improvements vary by modality: while chronic resistance training enhances sleep quality, its independent effects diminish when combined with aerobic exercise. Aerobic exercise optimizes sleep architecture through thermoregulatory and melatonin pathways [67]. Critically, sleep insufficiency reduces exercise motivation, increases fatigue perception, and impairs athletic performance—establishing a negative cycle that compromises exercise persistence. Furthermore, negative emotions constitute significant risk factors for sleep deterioration [68]. During such states, individuals exhibit markedly reduced exercise engagement, forming a secondary negative feedback loop.

These findings suggest that scientifically designed exercise programs may enhance sleep quality, potentially through mechanisms such as metabolic homeostasis regulation, though efficacy varies across exercise modalities. Specifically, resistance training yields less pronounced benefits than aerobic activities such as running and swimming. Universities should establish multidimensional health promotion systems integrating theoretical education with practical interventions. Theoretically, curricula must strengthen students’ cognitive awareness of schedule management, emotion regulation, and sleep hygiene. Concurrently, institutions should organize structured aerobic programs and sports events, enabling experiential recognition of exercise benefits to sustain engagement motivation.

### The mediating role of self-efficacy in consistent exercise and emotional regulation ability

This study demonstrates that self-efficacy mediates the relationship between consistent exercise and emotion regulation, aligning with prior evidence [69]. Notably, Chinese male college students exhibit higher self-efficacy, physical activity levels, and emotion regulation capacity than females [34], whereas women show stronger negative correlations between vigorous exercise and negative emotional states [70].

Social cognitive theory posits that physical exercise primarily enhances self-efficacy through achievement experiences, directly enabling positive behavioral predictions [71], This generalized self-efficacy promotes active emotional coping over avoidance behaviors [72]. During exercise, physiological responses like cardiorespiratory activation—though physiologically analogous to anxiety—are reinterpreted as “healthy bodily activation” through repeated exposure. By overcoming these responses, individuals cultivate confidence and attribute sensations to physiological rather than emotional causes [73]. Concurrently, exercise develops goal-setting competence, perseverance, and problem-solving strategies. These transferable skills strengthen emotional management neural circuits and enhance stressor control [74]. Crucially, while single exercise bouts yield transient mood benefits via physiological pathways, sustained exercise is required for enduring self-efficacy enhancement [75], Moreover, self-efficacy mediation necessitates moderate-to-vigorous intensity activity, with lower intensities showing no significant effects on self-efficacy or downstream emotion regulation [76].

The mediating function of self-efficacy is extensively documented in empirical research. Studies consistently establish its critical significance for effective emotion regulation. This mediation process demonstrates dependence on exercise intensity, modality, and duration, with notable gender differences observed. Crucially, only sustained moderate-to-vigorous physical activity enhances self-efficacy through neurobiological pathways, thereby improving overall emotion regulation capacity. Low-intensity or short-term exercise interventions show minimal association with self-efficacy enhancement or emotion regulation improvement.

### The chain mediating effect between sleep quality and self-efficacy

This study reveals that physical exercise enhances emotion regulation not only directly but more significantly through a chained pathway: sleep quality improvement → self-efficacy enhancement. Crucially, exercise-induced sleep benefits are moderated by self-control levels, with maximal effects observed in individuals with lower baseline self-control. Those with higher self-control exhibit inherently better sleep quality [77]. Furthermore, sustained exercise effectively reduces sleep disturbances and improves sleep efficiency [78], while increasing slow-wave sleep duration [79]. The improvement in sleep, particularly the increase in slow-wave sleep, facilitates critical restorative functions. On one hand, it facilitates physiological restoration, including bodily tissue repair and energy homeostasis [79]. On the other hand, it is crucial for neurocognitive restoration, which involves the consolidation of memories and the replenishment of executive control functions mediated by the prefrontal cortex [21,80].

Sleep deprivation impairs emotion regulation capacity and amplifies negative affect [81], including significant executive function decline [80,82], Insomnia exhibits high comorbidity with depression and anxiety. Chronic sleep insufficiency induces cognitive errors and inefficiency, wherein failure experiences directly diminish self-efficacy through eroded confidence. Conversely, low mood states not only reduce self-efficacy but subjectively prolong sleep onset latency [21], further compromising sleep quality. Both physiological states and emotional conditions constitute critical sources of efficacy beliefs. Persistent negative emotions trigger diminished self-awareness, psychological depletion, and negative expectancy formation. During fatigue states, physiological signals amplify perceived task difficulty, even for simple tasks, generating “inability to cope” cognitions that reduce task initiation motivation and undermine persistence expectations, ultimately reinforcing negative affect.

Empirical evidence confirms that self-efficacy interventions effectively reduce presleep cognitive arousal and anxiety, thereby improving sleep quality [83]. Consequently, physical exercise, sleep quality, self-efficacy, and emotion regulation demonstrate interconnected relationships with mutual reinforcement effects, validating this study’s Hypothesis H1. These correlations manifest through tightly interwoven neurocognitive, psychophysiological, behavioral, and cognitive pathways. For optimal performance in physical exercise, academic, or professional domains—and for maintaining mental health—quality sleep and robust self-efficacy constitute equally essential, interdependent pillars: the former establishes neurophysiological foundations, while the latter provides psychological impetus for challenge engagement.

This study empirically validates the chain mediation of sleep quality and self-efficacy in the pathway from consistent exercise to enhanced emotion regulation among college students. Specifically, regular exercise improves sleep quality—particularly through increased slow-wave sleep duration—facilitating physiological restoration and neural optimization. This neurophysiological foundation attenuates emotional volatility and stress perception during sleep. Concurrently, improved sleep bolsters positive self-perception, strengthening confidence in challenge engagement, exercise persistence, and goal attainment. The synergy between physiological stability and augmented self-efficacy promotes proactive cognitive reappraisal strategies for stress management, ultimately enhancing overall emotion regulation capacity and confirming Hypothesis H3. Universities should prioritize optimizing circadian rhythm management systems, minimizing social activity disruptions to sleep patterns. This consolidates exercise-induced slow-wave sleep enhancement and establishes physiological foundations for emotional stability. Concurrently, integrate emotion management training, sport psychology, and health science modules into curricula to teach scientific regulatory strategies—particularly cognitive reappraisal—while fostering evidence-based understanding of exercise benefits. To sustain exercise adherence, complement physical practice with professional psychological support services that enhance perceived control over athletic performance and cultivate positive affective experiences during sports participation.

### Limitations

This study has several limitations. Firstly, the cross-sectional design precludes causal inferences between variables, requiring future longitudinal investigation. Secondly, the generalizability of our findings may be limited by the regional focus on Henan Province. Although Henan is a major central Chinese province with a large population and diverse socioeconomic tiers, which strengthens its internal representativeness, cultural, educational, and lifestyle differences across China could influence the observed relationships. For instance, students in coastal, more developed regions might have different exercise facilities, academic pressures, or sleep habits that could moderate the strength of the chain mediation pathway identified in this study. Therefore, while the core psychological and physiological mechanisms are likely applicable, the specific effect sizes and the relative importance of each path might vary in different regional contexts. Future research should validate this model in other geographic areas to establish its broader applicability. Third, incorporating additional mediators—such as social support and stress coping styles—would strengthen the theoretical model’s comprehensiveness. Fourth, the overrepresentation of freshmen (72.6%) in our sample limits the generalizability of the findings to senior students, who face distinct pressures. A more balanced sample in future work is needed to enhance external validity.

## Conclusions

Exercise Adherence directly enhances college students’ emotion regulation capacity while also exerting indirect effects through two distinct pathways: independently via sleep quality and self-efficacy, and jointly through their chain-mediating relationship. This triple-path mechanism systematically elucidates how physical exercise improves emotion regulation.

## Supporting information

S1 Data (XLSX)

## Abbreviations

EAS: Exercise Adherence Scale
EIS: Emotional Intelligence Scale
PSQI: Pittsburgh Sleep Quality Index
GSES: General Self-Efficacy Scale
MVPA: Moderate-to-vigorous physical activity
EA: Exercise Adherence
SQ: Sleep Quality
SE: Self-Efficacy
ER: Emotional Regulation
